# RT-QuIC: a highly promising diagnostic method for neurodegenerative diseases—advantages and limitations

**DOI:** 10.3389/fneur.2025.1578252

**Published:** 2025-05-27

**Authors:** D. Koníčková, D. Hraboš, K. Menšíková, L. Tučková, M. Kaleta, M. Strnad, C. Colosimo, P. Kaňovský

**Affiliations:** ^1^Department of Neurology, Faculty of Medicine and Dentistry, Palacky University, Olomouc, Czechia; ^2^Department of Neurology, University Hospital Olomouc, Olomouc, Czechia; ^3^Department of Molecular Pathology, Faculty of Medicine and Dentistry, Palacky University, Olomouc, Czechia; ^4^Olomouc Brain Bank, Department of Neurology and Department of Clinical and Molecular Pathology, University Hospital Olomouc and Faculty of Medicine and Dentistry, Palacky University, Olomouc, Czechia; ^5^Laboratory of Growth Regulators, Institute of Experimental Botany of the Czech Academy of Sciences & Palacky University, Olomouc, Czechia; ^6^Department of Neurology, Santa Maria University Hospital, Terni, Italy

**Keywords:** alpha-synuclein, seed amplification assays, RT-QuIC, Parkinson’s disease, biomarkers

## Abstract

Although it has been more than 200 years since Parkinson’s disease was described, we have not established biomarkers for its definitive diagnosis yet. Moreover, there is a similar case for the entire group of α-synucleinopathies, which are all characterized by the pathological accumulation of aggregated α-synuclein (α-Syn) in the brain and other tissues. In different biological materials (blood, cerebrospinal fluid, saliva, or skin), α-Syn exists in various conformations and various aggregated states depending on the surrounding environment. Lewy bodies have been considered a pathognomonic feature of Parkinson’s disease for over 100 years, and α-Syn has been known to be a key component of Lewy bodies for over 25 years, making it possible to confirm the diagnosis by *post-mortem* examination of brain tissue. To overcome these limitations, novel analytical seed amplification assays (SAAs) were introduced, and they quickly became one of the most effective diagnostic tools for *antemortem* detection of α-synucleinopathies. As they require minimal sample amounts to provide consistent, rapid, and reliable results, SAAs are ideally suited for biomarker determination. This review examines SAA analytical and detection methods, their advantages and strengths, as well as their limitations and shortcomings that need to be addressed to establish a reliable and reproducible protocol. This could serve as a diagnostic methodology worldwide to determine the presence of pathological α-Syn protein at early stages and help develop effective disease-modifying treatment.

## 1 Alpha-synuclein: structure and function

Alpha-synuclein (α-Syn) is a highly conserved small acidic protein with a molecular mass of 14 kDa, predominantly found in presynaptic terminals of neurons. It consists of three basic domains: an amphiphilic N-terminal region [NT; amino acid residues (AAA) 1–60], a central hydrophobic non-amyloid component domain (NAC; AAA 61–95), and an acidic C-terminal domain (CT; AAA 95–140), which contributes to calcium binding and chaperone-like activity and play the role of a chaperone (regulation of synaptic vesicle binding, suggesting the physiological role for α-Syn in the membrane) (1). In its natural state, α-Syn is natively unfolded and intrinsically disordered protein, but could potentially adopt helical conformations upon membrane binding (2, 3). While its exact biological role remains not fully understood, α-Syn is believed to regulate synaptic vesicle trafficking and neurotransmitter release (4).

### 1.1 Alpha-synuclein: aggregation, seeding and prion-like spreading

Under pathological conditions, α-Syn can self-aggregate, potentially propagate in a prion-like manner, and accumulate into tetramers ~58 kDa. The imbalance between the monomeric and tetrameric states makes it prone to conformational changes, forming beta-folded sheet-rich structures, typical of amyloid fibrils (1, 5, 6). Altered α-Syn with beta-sheet aggregates converting them into abnormally aggregated non-fibrillar and fibrillar forms. The as-of-yet poorly understood seeding process leads to the fibrillization of oligomers, with seeding-efficient forms often being small fibrillar aggregates (~50 nm) (7). Oligomers are toxic, causing cellular dysfunction, including synaptic, mitochondrial, lysosomal, and autophagic impairments, and can form pores in cell membranes increasing its permeability (1).

α-Syn is proposed to spread in the nervous system in a prion-like manner, although the molecular pathways of seeding, exocytosis, and interaction with extracellular components are unclear. Misfolded α-Syn seeds are secreted into interstitial compartment, either as free protein or in vesicles, and spread rapidly through neurons, aggregating in affected cells (1). Furthermore, aggregates of α-Syn are detected in extracellular spaces, they circulate in biological fluids (CSF, blood) and could be detected in biological samples other than the central nervous system (skin, salivary and lacrimal glands, intestinal mucosa). The process of protein misfolding, aggregation, and spreading appears to begin decades before the onset of clinical symptoms (8, 9).

Interestingly, α-Syn does not always aggregate and spread in the same manner. It is currently almost certain that the aggregated form α-Syn may have different conformational structures, usually referred to as conformational strains, which probably affect various brain regions and lead to different clinical phenotypes (5).

## 2 Alpha-synuclein and neurodegeneration

**Figure 1:**
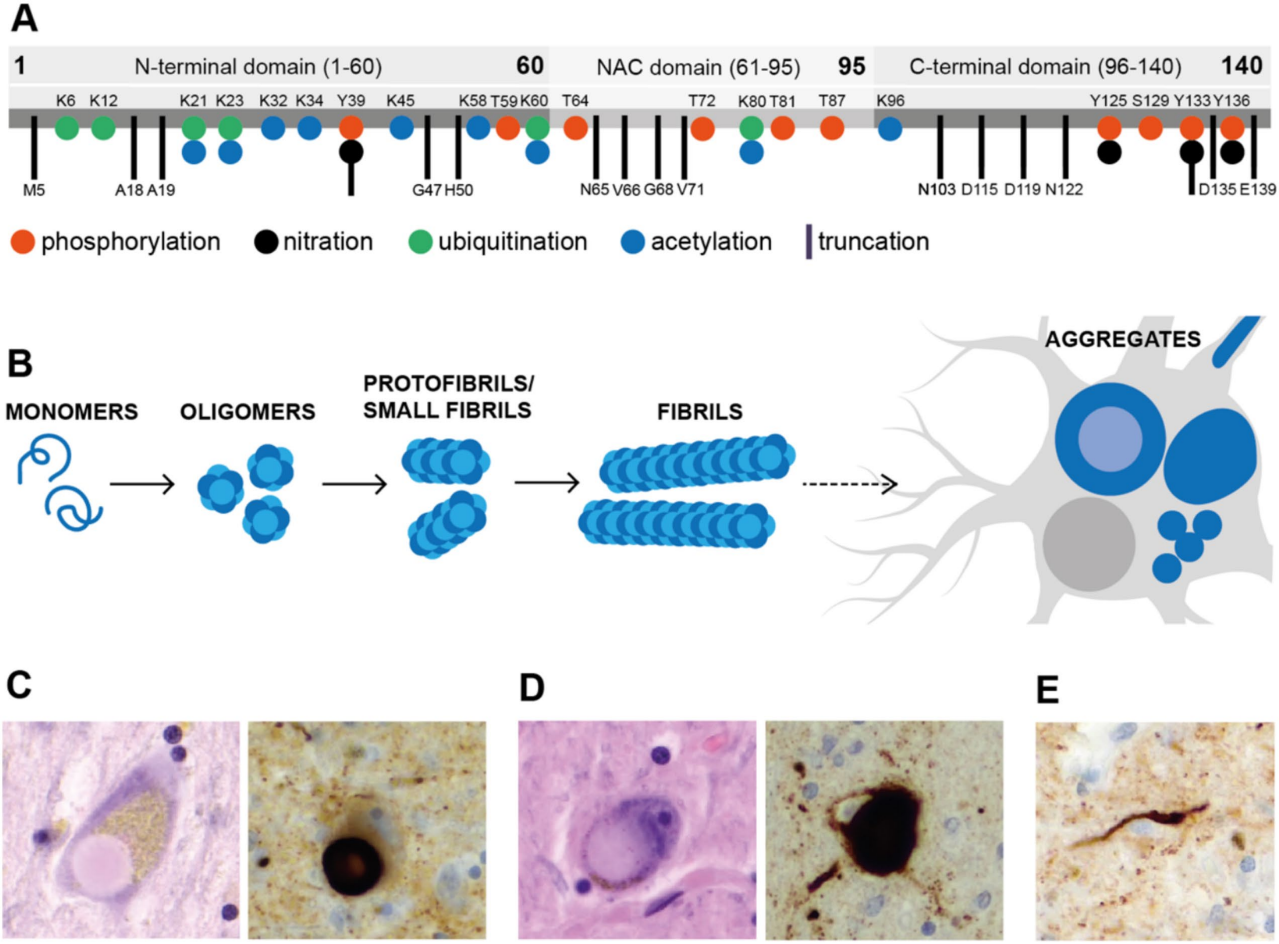
The most common post-translational changes and aggregates of α-synuclein (α-Syn). The most common post-translational changes of α-Syn include phosphorylation, nitration, ubiquitination, acetylation and truncation of the polypeptide chain **(A)**. Apart from these, changes like glycation and SUMOylation occur as well. The process of protein aggregation is complex **(B)**. Monomeric α-Syn tends to form oligomers and long amyloid fibrils; the pathway between these and microscopic aggregates like Lewy bodies, however, is not clearly defined. Cortical Lewy bodies are round complex aggregates with peripheral halo (**C**, 400×). Pale bodies are considered to be precursors of Lewy bodies (**D**, 400×). Similarly, aggregates form in neurites as well and are called Lewy neurites (**E**, 400×). **(C–E)** Shows representative microphotographs of aggregates in Parkinson’s disease-affected human mesencephalon in hematoxylin/eosin staining and immunohistochemistry analysis (anti-alpha-synuclein antibody clone 4D6).

Misfolded α-Syn can act as a seed that induces the conversion of normal cellular α-Syn, allowing the transcellular spread of Lewy bodies (LB) pathology in a prion-like manner (10). The aggregation of α-Syn follows a seeding/nucleation mechanism that is characterized by the progressive formation of oligomers, protofibrils, and fibrils that culminate in LB or glial cytoplasmic inclusions (GCIs). In the seeding/nucleation polymerization reaction, the first and rate-limiting step is the formation of a nucleus, which then forms large aggregates at the expense of the monomeric protein. The nucleus can serve as a seed to catalyze further polymerization, most likely remaining misfolded to the protein oligomer, which can be highly neurotoxic even before it grows into larger aggregates. A typical feature of the seeding/nucleation process is that adding preformed seeds can significantly accelerate the aggregation process by scooping soluble native protein into the growing aggregate. The seeding/nucleation mechanism provides a biochemical explanation for the propagation of misfolded proteins between cells and tissues. The molecular mechanisms of aggregate formation in the prion protein and α-Syn are similar. This suggests that misfolded proteins can spread between cells and tissues to initiate and propagate pathogenesis. A substantial body of evidence from the last decade demonstrates that misfolded α-Syn aggregates can move from cell to cell, causing damage from one brain region to another (4). The formation of LBs is extremely complex and involves the accumulation of fibrils, post-translational modifications, and interactions between α-Syn aggregates and membranous organelles (Figure 1) (11).

Lewy body diseases (LBDs) in the brain most commonly begins in the medulla oblongata, spreading intracerebrally along an ascending pathway from the brainstem to the cerebral cortex, traversing limbic regions. The staging scheme proposed by Braak et al. (12, 13) recognizes six pathologically distinctive stages, progressively affecting the nervous system in a caudal-to-rostral direction over the course of the disease. It begins in the peripheral autonomic neurons and olfactory regions (Braak stage 1). Then, it continues to spread in the lower brain stem (Braak stage 2), extends to the midbrain, forebrain, and limbic system (Braak stages 3–4), and finally progresses to primary and association cortical areas and insular cortex (Braak stages 5–6). Braak staging has been, with some reservations, widely accepted, thanks to its capacity to partially explain the temporal progression of both motor and non-motor clinical symptoms of PD.

However, there are well-known exceptions to the progression of LB pathology described above (14–17), the most common of which is the amygdala-predominant variant in which there is extensive α-Syn deposition in the amygdala associated with only mild and localized LB pathology in the brainstem. After stratification by extent and burden of LB pathology, significant differences in test sensitivity were observed. Performance against the gold standard (neuropathology) represents a key issue in the validation of new pathology-specific biomarkers. Such studies are severely limited by the latency between *in vivo* and *post-mortem* assessment, especially for typically long-term pathological processes. The presence of LB pathology within the spinal cord and/or peripheral ganglia, or CSF contamination favoring spontaneous aggregation of recombinant α-Syn, may explain these discordant positive results. Further studies in larger sample groups are needed to address this critical issue further (10).

## 3 Alpha-synuclein detection

Apart from standard biochemical assays (Western blot, ELISA, etc.) for α-Syn in blood or CSF samples, new analytical detection methods, characterized by high sensitivity (90%) and specificity (82–100%) of in-sample protein detection are emerging (18, 19). The best-known of these are (1) seeded aggregation assay (SAA) and (2) immunohistochemical analysis of pathological α-Syn in the peripheral nervous system (20).

SAA methods, also known as misfolded cyclic protein folding amplification (PMCA), including the more recent RT-QuIC method, amplify small amounts of aggregated protein in body fluids or tissue homogenates in an interactive process of aggregation and partial disaggregation (21).

The detection of pathological α-Syn by seed amplification assays (SAA) has emerged as the central method discussed in this review. Therefore, other techniques, such as immunohistochemical analyses, are not further detailed.

## 4 Seed amplification assay

Seed amplification assay (SAA) is a novel *in vitro* approach that exploits the polymerization properties of prion amyloidogenic proteins for their ultrasensitive detection in biological fluids and tissues (22).

SAAs have been recently introduced in α-synucleinopathies, including PD, to detect disease-specific misfolded α-Syn in biological samples. Qualitative dichotomous seeding response (+/− seeding) is valuable to stratifying and enriching cohorts for α-Syn pathology. In general, more quantitative parameters related to dynamic disease progression and phenotypic trajectories may serve as exploratory outcome measures for α-Syn. Differences in seeding are associated with conformational heterogeneity of the seed. Posttranslational modifications of the α-Syn protein (phosphorylation, nitration, various types of mono- or polyubiquitination, truncation SUMOylation and O-GlcNacylation), possibly caused by differences in cellular pathways induced by different α-Syn strains, could modulate their pathogenic effects by affecting physicochemical properties (23).

CSF α-Syn SAAs have demonstrated high diagnostic performance in confirming LB pathology in the majority of patients with prodromal LBD symptoms and even in asymptomatic patients. Together with the CSF analysis data, the results obtained by performing α-Syn SAA on brain homogenates from different regions suggest that the pathological burden of LB is the primary factor influencing the sensitivity of the test (10). In this context, the kinetic parameters of the SAA response, such as the lag phase (LAG) (the time required to reach seeding), the fluorescence peak response (F_Max_), and the area under the fluorescence signal curve (AUC) are candidates for evaluation in individuals who exhibit positive α-Syn seeding (23). In addition, the protocols used also differ in several factors such as pH of the reaction buffer, incubation, presence of detergents, and type of plate used that affect the aggregation response and sensitivity of α-Syn SAA. The lack of uniformity of recombinant α-Syn protein (in-house prepared or commercially available) (20) presents a challenge for protocol standardization and interlaboratory reproducibility. As a result, most protocols are performed in-house (1). Based on the study findings, it is hypothesized that the pronounced kinetic profiles of α-Syn seeding in CSF are associated with a more rapid progression to clinical disease milestones such as cognitive impairment. A high number of positive replicates and a more pronounced kinetic profile of α-Syn SAA, as manifested by a shorter LAG, higher F_Max_, and higher AUC, is associated with a high incidence of cognitive impairment and a shorter duration of cognitive development (23). In addition, kinetic analysis of the fluorescence signal suggests that the aggregation response in α-Syn SAA tends to depend on the seed concentration phenotype, and may provide quantitative information, with LAG and the number of positive replicates being the most promising and reliable variables for prospective development (1, 10).

The results of these studies suggest that since α-Syn SAA has been successfully used to detect prodromal symptoms prior to clinical diagnosis, it may also be used to detect presymptomatic and preclinical stages of the disease. Future research will aim to demonstrate that α-Syn SAA can provide quantitative information on the number of seeds in a biological sample and determine whether it correlates with the disease stage, which can be used to monitor treatment efficacy. However, it is unclear whether the concentration of misfolded α-Syn correlates with disease progression. Based on the available evidence, it is most likely that the misfolded protein is the critical event that initiates the pathological cascade in the brain, and once this process begins, the amount of protein aggregates that accumulate in the brain may not correlate with the pathological damage process (24).

The methodology encounters several problematic issues that could affect SAA analysis, its reproducibility and diagnostic accuracy—the presence of post-translational modifications that are not present in the recombinant α-Syn protein used as a substrate; other brain cofactors that may be involved in the propagation of α-Syn pathology *in vivo* and, thus, with the absence of these other cofactors, the *in vitro* reaction may not successfully replicate the properties of endogenous α-Syn aggregates.

Future efforts are needed to standardize materials, including recombinant α-Syn as a substrate, and to standardize protocols for RT-QuIC (1).

### 4.1 Real-time quaking-induced conversion

**Figure 2:**
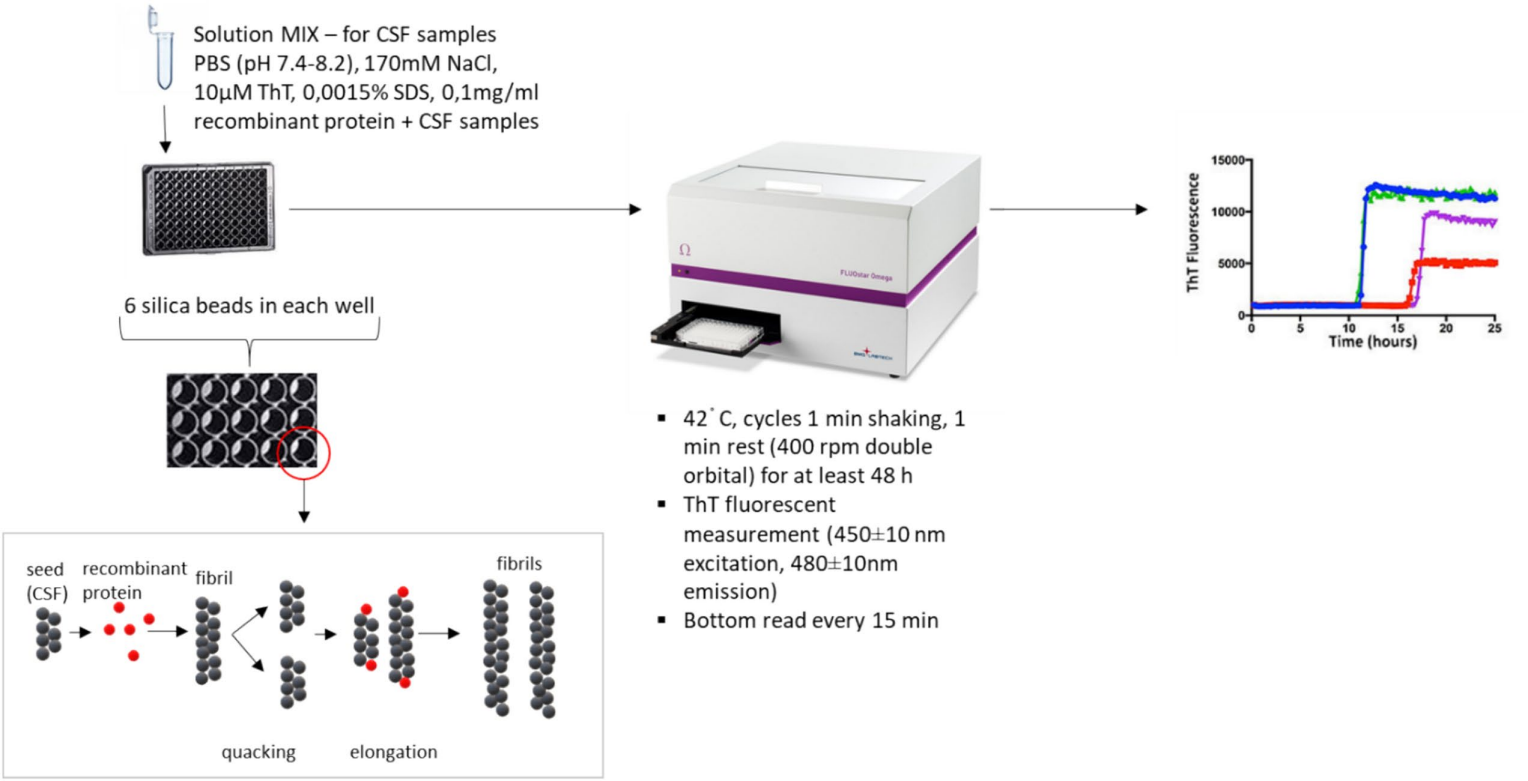
Schematic representation of the RT-QuIC method. The prepared reaction mix (85 μL) is pipetted together with the sample (15 μL) into a black 96-well plate with a transparent base, in which six silica beads are inserted into each well, during which the fusion reaction of native α-Syn with the recombinant protein occurs, the fragmentation and extension of the newly formed fibrils occur within the analytical steps of shaking and standing; the plate is sealed with foil and inserted into a FluoStar Omega (BMG LABTECH) before analysis. The fluorescent detection signal is collected every 15 min for at least 48 h, allowing us to read the fluorescent absorbance in real time.

Real-time quaking-induced conversion (RT-QuIC) is a widely used SAA that allows the efficient detection of small amounts of protein (α-Syn aggregates) in body fluid or tissue samples. The system is initiated by a biological sample in which pathological α-Syn aggregates are induced by aggregation of a recombinant α-Syn substrate, and the ability of the pathogenic protein to induce a conformational change in the normal protein is detected. The pathogenic protein thus acts as a “seed” for substrate conversion. RT-QuIC allows specific detection of atto- or femtogram quantities of proteopathogenic seeds in biological samples, opening a better route to *intra vitam* diagnostics (6). It has been shown that α-Syn spreads similarly to prions, via conformational conversion and fibril formation (25). Amyloid α-Syn protofibrils tend to aggregate and serve as a template to convert normal endogenous α-Syn into abnormal proteins, thereby amplifying them. This process allows the amplification of traces of amyloid fibrils in patients’ biofluid samples, and the presence of amyloid fibrils is revealed by real-time aggregation kinetics using fluorescence that couples to the beta-sheet structure of α-Syn aggregating proteins (binding of the protein to the fluorescent dye shows enhanced fluorescence activity) (Figure 2) (26).

#### 4.1.1 RT-QuIC parameters

**Figure 3:**
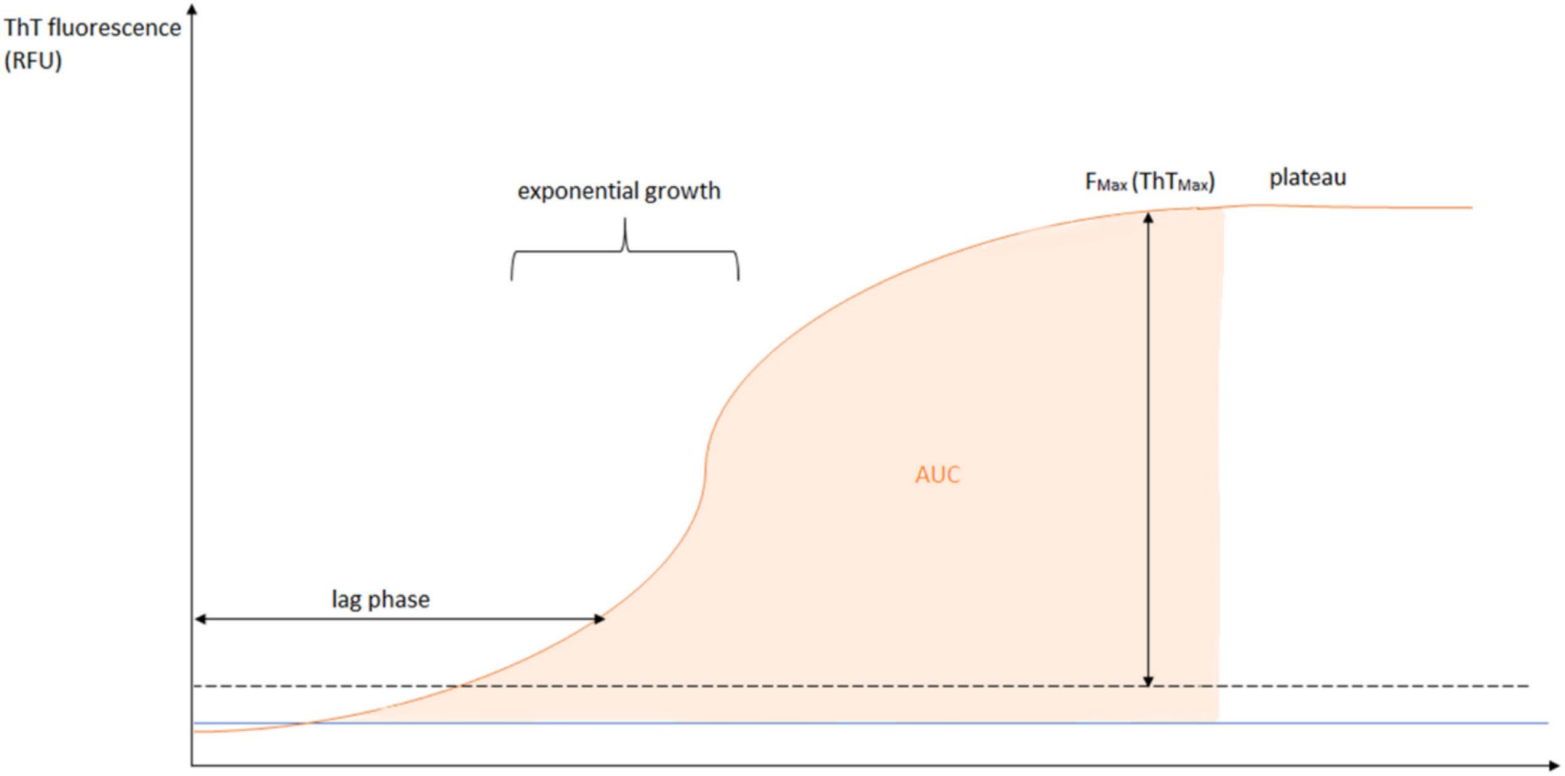
Schematic representation of parameters of the RT-QuIC method on the fluorescence axis plotted against time. The blue line represents the negative control. The orange line represents the sample/positive control. The given curve shows the parameters: the lag phase (the time needed to reach the seeding), the F_Max_ (fluorescence peak response), the AUC (area under the fluorescence signal curve) and the plateau phase, where the curve no longer rises.

The amplification of α-Syn seeds in RT-QuIC is cyclic. Initially, the aggregates are fragmented into smaller seed self-precursors by a fragmentation step. These smaller seeds are progressively elongated by a recombinant protein, thereby increasing their number (number of seeds in the sample) (Figure 2). Theoretically, a larger number of seeds results in faster aggregation (27). The time to reach 50% of the fluorescence maximum (T_50_) is the best kinetic parameter that correlates with seed concentration. The relative fluorescence intensity (RFU) reflects the real-time amplification efficiency. Other aforementioned kinetic parameters, such as protein aggregation rate (PAR), LAG, F_Max_, and AUC, can be used to evaluate the amplification efficiency. The amplification allows the detection of seeds using specific fluorescent dyes such as thioflavin T (ThT), which binds specifically to amyloid structures (Figure 3) (27).

Semi-quantitative RT-QuIC parameters may indicate potential differences between strains, as different conformations of the same pathogenic seed could exhibit differences in promoting aggregation. Different results may be observed when comparing two pathogenic seeds. It is possible that one seed may be faster in inducing aggregation of its substrate, resulting in a shorter LAG. A similar result could be obtained with the same seed applied at different concentrations, highlighting the importance of seed quantity. Although they have similar maximum intensities, the two predicted strains can be distinguished by both the LAG and the AUC. In another case, the LAG may be identical, but the strain can be identified by the lower F_Max_ and therefore lower AUC. This may reflect a reduced ability of the seed to bind substrate, resulting in weaker aggregation (28). This can be illustrated by the case described by Groveman et al. (27), where reactions with PD seed showed a lower F_Max_ signal during the run compared to reactions related to dementia with Lewy body (DLB) seed. This result could also be obtained with seeds with lower avid binding to the detection dye, as reported by Ferreira et al. (29). The most complex scenario involves seeds with similar latency and avidity for the substrate (LAG and maximum intensity, respectively) but different exponential phases. A steeper curve would indicate a greater ability to convert the substrate once the reaction is initiated. In this case, although the maximum intensity and LAG are similar, the two strains can be distinguished by the value of the AUC, with the steeper signal resulting in a larger area. Finally, a seed may support conversion faster than another but exhibit lower F_Max_. This case is similar to the observation reported by Shahnawaz et al. (8) and Poggiolini et al. (26), where seeds derived from multiple-system atrophy (MSA) showed a faster but less intense ThT-based signal compared to PD-derived seeds (28).

However, the RT-QuIC assay parameters do not only correlate with the number of seeds in the samples but also with the internal ultrastructure of α-Syn aggregates (30). Although RT-QuIC shows high sensitivity and specificity in detecting α-synuclein seeds, its ability to reliably distinguish PD from other α-synucleinopathies like MSA or DLB remains limited, especially in atypical cases. Structural differences in α-synuclein aggregates, such as the distinct conformation of MSA-derived seeds, can lead to reduced detection in standard ThT-based assays, increasing the risk of false negatives. Additionally, co-pathologies common in neurodegenerative diseases may cause false positives, as SAA detects α-synuclein presence but not disease-specific patterns. For example, incidental Lewy body pathology (iLBD), which occurs in approximately one in six older adults without clinical signs of PD, can yield positive RT-QuIC results and thus confound diagnostic interpretation (31). Furthermore, SAA positivity has been reported in asymptomatic carriers of LRRK2 mutations and individuals with PRKN mutations, despite a reduced prevalence of Lewy pathology in postmortem studies of these genetic forms of Parkinsonism (32, 33). In LRRK2 PD specifically, SAA sensitivity is notably reduced (around 67.5%), and in normosmic female carriers, it drops below 15%, suggesting a potential disconnect between clinical phenotype and α-synuclein pathology. Such findings underscore the importance of considering clinical features, genetic background, and potential co-pathologies when interpreting RT-QuIC results. Assay variability across studies and lack of standardization further complicate interpretation. Therefore, RT-QuIC results should always be interpreted alongside clinical findings, genetic testing, and complementary biomarkers to improve diagnostic accuracy and reduce misclassification in atypical or genetically defined cases (34).

#### 4.1.2 Limits of RT-QuIC in the diagnosis of α-synucleinopathies

Although RT-QuIC has many advantages, it is still a novel and emerging method currently used mostly for research purposes. Understandably, there are some limitations in its use for α-synucleinopathy diagnostics.

1) Most importantly, there is a need for standardization of recombinant protein. There are a variety of protein forms, including wild-type and mutant ones (e.g., A53P, A30P). Each of them provides a different result.
2) RT-QuIC analysis has been successfully demonstrated in both CSF and peripheral tissues (skin, colon). However, there is a need for more studies utilizing available tissue obtained by minimally invasive procedures (35).
3) The process and results of RT-QuIC are influenced by a number of methodological factors, including shaking rate, oscillation interval, temperature, overall pH of the reaction system, and concentration of the denaturing agent.

The incubation/shaking cycle is a key factor for successful RT-QuIC. However, prolonged shaking increases the likelihood of spontaneous monomer aggregation, leading to false positive results. Incubation is an important step because it influences the interaction between seeds and substrate to expand fiber aggregation. The shaking frequency affects the reactivity of RT-QuIC, as studies have shown that adjusting the shaking frequency from 700 rpm to 1,000 rpm causes a delayed phase, which can be effectively shortened and then the signal repeatability is improved (36). Temperature also affects the RT-QuIC procedure. For example, increasing the temperature from 42°C to 60°C increases the response efficiency and shortens the lag time (37). However, the excessive temperature can lead to the formation of more aggregates or adsorption on the grooved surface, reducing the surface area for ThT binding, and thus decreasing ThT fluorescence values. In most studies, the temperature is set at 42°C, since it provides the best balance between the lag phase, the period of prolonged protein aggregation, and the rate of sample evaporation. The pH also plays a role in RT-QuIC. Low pH promotes the aggregation of misfolded proteins by increasing their hydrophobicity (38). The concentration of SDS can also make a difference, as the presence of SDS can result in larger and coarser fiber aggregates (39). Another critical issue involves the source of recombinant α-synuclein. While many laboratories synthesize recombinant α-synuclein in-house, this procedure is technically challenging, time-consuming, and may lead to batch-to-batch variability. Increasingly, commercially available recombinant α-synuclein products are being adopted to improve standardization and reproducibility across studies.

#### 4.1.3 Assessment of method reproducibility; pre-analytical optimization

Assessing the reproducibility of the qualitative and quantitative data provided by the RT-QuIC method is another unresolved issue that severely limits its impact in clinical practice. Quantitative SAA could contribute to the assessment of disease severity and progression.

Among the most recent studies, Mammana et al. (22) provided data on the pre-analytical preparation of RT-QuIC runs. Firstly, the effect of repeated thawing and freezing of CSF samples was tested for the first time. In contrast to standard biochemical tests, the RT-QuIC did not affect the test results with repeated cycles of sample thaw, the samples remained consistently positive or negative, and the kinetic parameters of the fluorescence curve did not differ significantly (kinetic parameters were not altered by freezing/thawing for up to 7 cycles).

Comparison between non-centrifuged and centrifuged CSF samples revealed that uncentrifuged CSF stored at 4°C exhibited significantly shorter lag times (LAG) and higher maximum fluorescence (F_Max_), enhancing seeding detection efficiency. Another examined parameter was the addition of non-ionic detergents to freshly collected CSF, which resulted in a shorter LAG, lower F_Max_, and less prolonged plateau phase, as reflected by a large change in response kinetics. Notably, the addition of non-ionic detergents also affected the kinetics of the negative samples, resulting in false positive curves. The results of this study suggest that increasing the number of replicates to eight slightly improves the accuracy of the assay. In addition, the authors found that the number of positive replicates in undiluted CSF samples was positively associated with the likelihood of positive RT-QuIC results with increasing sample dilution, further supporting the correlation between the number of positive replicates and the amount of α-Syn in CSF.

Blood contamination in CSF samples was the last studied parameter (22). From the results of the study, it is evident that blood contamination in CSF higher than 0.1% significantly inhibits the RT-QuIC response (contamination up to 0.001–0.01% shows longer LAG and lower F_Max_ but does not inhibit the response). Therefore, the authors recommend avoiding the first 2 mL of collected CSF, where blood contamination is most likely to occur.

Our experience confirms that the pre-analytical part, the protocol setting of the methodology, is a very demanding and complicated process that depends on multiple factors and their mutual combination. The importance of the analytical part lies not only in the individual steps of sample processing but also in the times of thawing the sample/recombinant protein and its subsequent dissolution. Given that α-Syn tends to aggregate spontaneously, it is also necessary to shake/vortex/sonicate the samples several times during pipetting to obtain the most homogeneous sample possible. The freshness of the solutions and the protein concentration in the sample are also very important factors.

**Figure 4:**
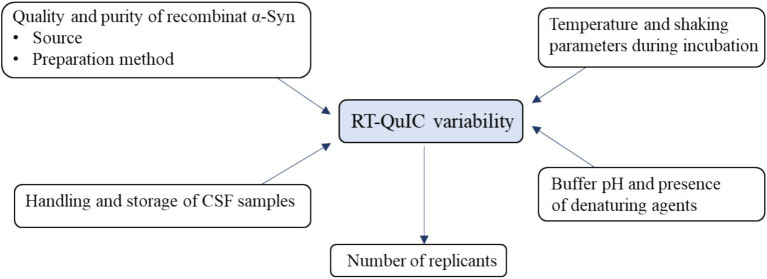
Schematic representation of key pre-analytical and analytical variables affecting RT-QuIC performance.

To assist researchers and ensure reproducibility of RT-QuIC assays, a schematic summary of critical factors that introduce variability is provided in Figure 4.

To enhance reproducibility and reliability across laboratories, the following best practices are suggested:

- Freshly prepared aliquots of recombinant α-synuclein should be used, and multiple freeze-thaw cycles should be avoided.
- Uncentrifuged CSF samples kept at 4°C should be preferred to achieve shorter lag phases.
- The first 2 mL of collected CSF should be discarded to minimize blood contamination.
- A shaking frequency of ~700 rpm and an incubation temperature of ~42°C should be maintained.
- Buffer composition, especially pH and detergent concentration, should be standardized.
- The number of replicates (at least 6–8) should be increased to improve diagnostic reliability.
- Plates should be carefully sealed to prevent evaporation and sample degradation.

#### 4.1.4 RT-QuIC as a clinical diagnostic tool

RT-QuIC for the detection of misfolded α-Syn associated with LB has become a laboratory tool with huge diagnostic potential for a highly prevalent group of α-synucleinopathies (22). Currently, studies show that the RT-QuIC assay can successfully amplify and detect α-Syn in the CSF and skin samples from patients with synucleinopathies, with the minimum limit in the attogram to femtogram range of protein per sample, supporting its potential for early clinical detection (30, 38). There is still a lack of in-depth knowledge on the impact of pre-analytical and analytical factors that can significantly influence the assay results. Therefore, systematic identification of those factors that potentially influencing α-Syn RT-QuIC data may improve the standardization of processing protocols between laboratories and consequently the reproducibility and clinical value of this already robust assay. RT-QuIC results are currently being analyzed to provide a qualitative answer (yes/no) with high sensitivity and specificity, as this methodology is much more sensitive than the conventionally used ELISA, detecting only those forms of α-Syn that are capable of undergoing the protein misfolding (“permissive templating”) that is key to disease pathogenesis (1, 25, 26). Nevertheless, a growing body of evidence shows that analysis of RT-QuIC reaction kinetic curves can also contribute to quantitative data that indirectly reveal the number of protein seeds in biological samples and possibly the pathological burden of LB (22, 40).

A substantial body of evidence has confirmed the high diagnostic accuracy of RT-QuIC across different sample types. In CSF, RT-QuIC shows a sensitivity of 90–95% and specificity around 90–98% for detecting PD and DLB (10, 22). Studies on skin biopsies report sensitivity between 80–90%, suggesting the usefulness of less invasive sampling (41). These results highlight RT-QuIC’s promising role in the early diagnosis of synucleinopathies, although further standardization efforts are needed for widespread clinical application (see Table 1).

**Table 1:** Diagnostic accuracy of RT-QuIC across different tissue types and clinical cohorts.

Tissue/sample type	Disease vs. control	Sensitivity (%)	Specificity (%)	References
CSF	PD vs. controls	89–97	90–100	(30)
	DLB vs. controls	95	96	(17)
Brain homogenate	LB stage vs. Braak pathology stage	70–90	80–100	(9)
Skin biopsy	PD vs. controls	95	100	(32, 33)
	DLB vs. PD	100	23	(9)
Olfactory mucosa	PD vs. controls	69–76	100	(35)
	MSA vs. controls	90	100	(35)
	DLB vs. controls	100	100	(36)

CSF, brain homogenate, skin and olfactory mucosa confirm its potential as a diagnostic tool in the early stages of the disease due to its high sensitivity and specificity for the detection of PD and other synucleinopathies.

#### 4.1.5 RT-QuIC and the skin

Several studies have demonstrated the presence of α-Syn in various sites of the peripheral nervous system (PNS), including the skin, gastrointestinal tract, olfactory mucosa, and submandibular glands. These findings suggest that the involvement of different parts of the PNS may occur simultaneously (42). Cutaneous α-Syn deposition in PD could be detected mainly in the autonomic nervous system.

Skin biopsy RT-QuIC may serve as a potential indicator of disease progression because it correlates with advancing disease stages and the presence of non-motor symptoms. Recent studies have also reported that skin RT-QuIC detected α-Syn misfolding with high sensitivity and specificity in identifying α-Syn aggregates associated with PD. Therefore, it can be considered a reliable and reproducible biomarker for PD (42). As in CSF, α-Syn seeding activity was detected in the skin by the RT-QuIC method. Disease duration was positively correlated with the final percentage of ThT fluorescence and negatively correlated with T_50_ and LAG. In addition, the Hoehn and Yahr Scale was correlated with the final percentage of ThT fluorescence. However, RT-QuIC scores were higher in patients diagnosed with non-motor symptoms such as REM sleep behavior disorder, constipation, or mild cognitive impairment (43).

#### 4.1.6 RT-QuIC and olfactory mucosa

Analysis of olfactory mucosa (OM) is a non-invasive method to detect α-Syn aggregates with considerable diagnostic robustness, providing much clearer results than CSF analysis alone. OM samples are likely to contain fewer proteins capable of cross-spreading α-Syn, thus increasing the efficiency of these samples. Importantly, OM is constantly regenerating, which affects the total amount of abnormally folded protein (pathological α-Syn). Recent studies have also shown that the relative combination of OM and CSF (44) and their relative levels of abnormally folded protein may also influence the results of RT-QuIC analysis (41). De Luca et al. (41) and Perra et al. (45) showed that α-Syn RT-QuIC detects α-Syn aggregates in the OM not only in patients with PD but also in patients with MSA and DLB. In addition, Perra et al. (45) provided preliminary evidence that the combination of OM and CSF can increase the agreement with clinical diagnosis by up to 100%. Remarkably, all patients with prodromal DLB had a positive OM RT-QuIC result, demonstrating that the α-Syn seed aggregation process can be detected during the early stages of the disease (45). Parallel studies focusing on the evaluation of mucosal biopsies were performed to identify aggregates of the pathological protein. However, no aggregates of α-Syn or phosphorylated α-Syn (p-α-Syn) were observed in patients, indicating the absence of LB. This may be explained by rapid olfactory cell turnover, with insufficient time to form LB. However, in contrast, other studies have shown that even in the absence of deposits, the OM tissue have seeding activity, suggesting that α-Syn is pathologically altered (46).

## 5 Conclusion

There is considerable interest in finding biomarkers of neurodegenerative diseases, including those that specifically target characteristically aggregated proteins. In addition to specificity, the ideal biomarker is highly advantageous if it supports early assessment of clinically relevant quantitative changes, has sufficient dynamic range in longitudinal studies, and can be measured in readily available biofluid or tissue samples (47). Further, useful biomarker could help to identifying and stratifying patient groups in the development of targeted therapies. The clinicopathological heterogeneity of PD and the limitations of current clinical-diagnostic criteria, especially early in the disease, highlight the need for pathology-specific biomarkers (23). Indeed, research has shown that the earlier treatment is initiated after PD diagnosis, the better is the prognosis (39). Undoubtedly, α-Syn analyzed by SAA/RT-QuIC may serve as a useful tool for the early detection of α-synucleinopathies (48). In addition, detection of p-α-Syn aggregates in autonomic nerve fibers, salivary glands, and skin *in situ* has emerged as a promising early biomarker as well. Importantly, these findings are not specific to LBD, as similar deposits can be seen in other α-synucleinopathies (47, 48).

Many questions remain unanswered regarding the use of RT-QuIC, and further improvements are necessary to enhance its effectiveness. Srivastava et al. (49) pointed out the considerable inter-assay variability in CSF samples, recommending a focus on mitigating and normalizing potential artifactual changes in seeding activity. Significant structural differences between human brain α-Syn fibrils and SAA fibrils, as well as pathological seeds *in vivo*, have been demonstrated in a recent study by Lee et al. (50). Taken together, these findings suggest that the biochemical profiles and structures of brain α-Syn fibrils vary between different synucleinopathies and individual patients, providing compelling evidence for the molecular diversity of these fibrils. Furthermore, as SAA fibrils do not adopt the same biochemical, structural and post-translational modification properties and showing the limitations of SAA in recapitulating the intrinsic biophysical properties of brain α-Syn fibrils. These results also highlight the need to re-evaluate the mechanism of SAA seeding and its ability to generate disease-relevant α-Syn fibrils in various *in vitro* and *in vivo* models. There is also an urgent need to further investigate α-Syn fibrils from different brain regions and peripheral sites to better understand α-Syn strains and their potential impact on pathological progression. Developing specific diluents to minimize variability in the seeds and matrices where they are located will likely be essential. Another challenge in using CSF as a biological material for diagnosis is the frequent reluctance of patients to undergo lumbar punctures, prompting the search for more accessible tissues and biofluids. Encouragingly, results from studies involving skin and OM support the notion that α-Syn could serve as a valuable and quantifiable biomarker in more accessible biomaterials. These findings show that such alternative biomaterials contain higher concentrations of α-Syn than those found in CSF, comparable to those in brain homogenates. This is due to the fact peripheral tissues (such as skin) can accumulate α-Syn deposits continuously for months to years, unlike CSF, which is replenished every 6–12 h. The extent to which peripheral α-Syn is present in CSF is unclear. However, the extent to which peripheral α-Syn deposits quantitatively correlate with disease duration, severity or various clinical parameters is also unknown and requires further investigation. Other important steps include the adaptation and validation of analytical and clinical technologies to detect α-Syn aggregates in biological fluids or tissues. It is also important to investigate whether analytical results can provide information about disease progression or identify individuals in the prodromal stage who are close to developing clinical symptoms, since in these early stages (asymptomatic patients), brain damage has not yet occurred and pathology in the brain is subtle. As other studies have shown, a faster gain response (in terms of time in hours) is associated with worse motor and cognitive disease progression (49).

Although it is challenging to set up these methods in different locations, as response parameters can vary significantly between laboratories, limiting the standardization of SAA, some published studies have quickly emerged and they are already being incorporated into routine worldwide. Nevertheless, there are several challenges and caveats, such as: quantification of results which are still largely dichotomous and variable (positive vs. negative, typically with three or four replicates per sample), indicating a different lag phase and lower minimum fluorescence. For this reason, it is recommended to combine SAA results with CSF biomarkers and use quantitative measurements to differentiate PD from MSA or other atypical parkinsonisms (51). One of the main challenges is the variability of test protocols and experimental conditions, which can lead to inconsistent results and hinder the reproducibility of findings at different stages. Standardization of assays and validation of results in large patient groups will be critical for the widespread transfer of SAA into clinical practice. The likely reasons for the inconsistent results that currently limit the “quantitative” utility of α-Syn-SAA may be the variability in reagents or techniques, the relatively small number of samples tested, or the intrinsic variability of the aggregation process itself. Another limitation of SAAs is their specificity for α-Syn pathology, which may limit their utility in diagnosing other NDs that share similar protein aggregation characteristics. Additionally, due to synuclein co-pathology or misdiagnosis, many patients may experience false-positive α-Syn SAA results (31). However, recent findings by Espay et al. (52) highlight that while α-Syn seeding is a biophysical process, it cannot ultimately clarify the origin or mechanisms of pathology in humans. Although SAA has been developed as a superior biomarker for detecting α-Syn pathology in individuals with clinical disease, it is not a comprehensive marker for unifying the diverse pathology of Parkinson’s disease (PD) or other synucleinopathies. While α-Syn SAA can reveal the presence of pathological α-Syn, it does not shed light on the underlying mechanisms that cause monomeric α-Syn to transition into its pathological form. Therefore, a positive α-Syn SAA can support the clinical diagnosis of PD but does not provide insights into disease pathogenesis, severity, progression rate, biological subtypes, or treatment responses. Although RT-QuIC currently holds significant promise as a supportive diagnostic tool, its routine clinical implementation will require several additional steps, including standardized assay protocols, multicenter validation studies, regulatory approval, and alignment with existing diagnostic frameworks. A clear roadmap for integration into clinical workflows and biomarker panels alongside clinical, imaging, and fluid-based biomarkers will be essential to fully realize its translational potential in the coming years. As highlighted by Grossauer et al. (53), promising results in alternative peripheral tissues—such as skin or olfactory mucosa - as well as in prodromal stages of synucleinopathies, further support the continued development of RT-QuIC toward routine screening and early detection strategies. These findings emphasize the need for harmonized protocols and further exploration of less invasive sampling methods to broaden clinical applicability and accessibility.

Future research efforts should focus on the development of seed aggregation assays that can detect a broader range of pathological protein aggregates, allowing for a more comprehensive and accurate diagnosis of NDs. An important future implication of research on seed aggregation assays research is the exploration of novel approaches to target and stop their spread in the brain. Early identification of patients may allow the slowing of progression through simple lifestyle changes or the use of safe pharmacological agents that can be administered to asymptomatic individuals. It will be also necessary to perform precise validation studies in multicentric cohorts comparing the assay utility in examining α-Syn levels in different tissues for instance CSF vs. skin, CSF vs. BS, CSF vs. OM and *vice versa.*
