# Correction: RT-QuIC: a highly promising diagnostic method for neurodegenerative diseases—advantages and limitations

**DOI:** 10.3389/fneur.2025.1636086

**Published:** 2025-06-09

**Authors:** D. Koníčková, D. Hraboš, K. Menšíková, L. Tučková, M. Kaleta, M. Strnad, C. Colosimo, P. Kaňovský

**Affiliations:** ^1^Department of Neurology, Faculty of Medicine and Dentistry, Palacky University, Olomouc, Czechia; ^2^Department of Neurology, University Hospital Olomouc, Olomouc, Czechia; ^3^Department of Molecular Pathology, Faculty of Medicine and Dentistry, Palacky University, Olomouc, Czechia; ^4^Olomouc Brain Bank, Department of Neurology and Department of Clinical and Molecular Pathology, University Hospital Olomouc and Faculty of Medicine and Dentistry, Palacky University, Olomouc, Czechia; ^5^Laboratory of Growth Regulators, Institute of Experimental Botany of the Czech Academy of Sciences & Palacky University, Olomouc, Czechia; ^6^Department of Neurology, Santa Maria University Hospital, Terni, Italy

**Keywords:** alpha-synuclein, seed amplification assays, RT-QuIC, Parkinson's disease, biomarkers

In the published article, there was an error in the Funding statement.

The author(s) declare that financial support was received for the research and/or publication of this article. This work was supported by grant from the Ministry of health, Czech Republic for the Conceptual Development of a Research Organization (FNOL, 0098892); IGA_LF_2024_021 and project “New Technologies for Digital Health” (Reg. No. CZ.02.01.01/00/23_021/0008829) from the Operational Programme Jan Ámos Komenský financed by the European Union (EU) and the State Budget of Czech Republic (AZV Grant No. NW25-04-00194).

The correct Funding statement appears below.

